# Use of Extended Characteristics of Locomotion and Feeding Behavior for Automated Identification of Lame Dairy Cows

**DOI:** 10.1371/journal.pone.0155796

**Published:** 2016-05-17

**Authors:** Gian Beer, Maher Alsaaod, Alexander Starke, Gertraud Schuepbach-Regula, Hendrik Müller, Philipp Kohler, Adrian Steiner

**Affiliations:** 1 Clinic for Ruminants, Vetsuisse-Faculty, University of Berne, Berne, Switzerland; 2 Clinic for Ruminants and Swine, Faculty of Veterinary Medicine, University of Leipzig, Leipzig, Germany; 3 Veterinary Public Health Institute, Vetsuisse-Faculty, University of Berne, Berne, Switzerland; University of British Columbia, CANADA

## Abstract

This study was carried out to detect differences in locomotion and feeding behavior in lame (group L; n = 41; gait score ≥ 2.5) and non-lame (group C; n = 12; gait score ≤ 2) multiparous Holstein cows in a cross-sectional study design. A model for automatic lameness detection was created, using data from accelerometers attached to the hind limbs and noseband sensors attached to the head. Each cow’s gait was videotaped and scored on a 5-point scale before and after a period of 3 consecutive days of behavioral data recording. The mean value of 3 independent experienced observers was taken as a definite gait score and considered to be the gold standard. For statistical analysis, data from the noseband sensor and one of two accelerometers per cow (randomly selected) of 2 out of 3 randomly selected days was used. For comparison between group L and group C, the T-test, the Aspin-Welch Test and the Wilcoxon Test were used. The sensitivity and specificity for lameness detection was determined with logistic regression and ROC-analysis. Group L compared to group C had significantly lower eating and ruminating time, fewer eating chews, ruminating chews and ruminating boluses, longer lying time and lying bout duration, lower standing time, fewer standing and walking bouts, fewer, slower and shorter strides and a lower walking speed. The model considering the number of standing bouts and walking speed was the best predictor of cows being lame with a sensitivity of 90.2% and specificity of 91.7%. Sensitivity and specificity of the lameness detection model were considered to be very high, even without the use of halter data. It was concluded that under the conditions of the study farm, accelerometer data were suitable for accurately distinguishing between lame and non-lame dairy cows, even in cases of slight lameness with a gait score of 2.5.

## Introduction

Lameness in dairy cows is an expression of pain [1,2] due to pathologies involving the locomotor apparatus. It causes high economic losses mainly due to decrease of milk yield and reduced fertility [3,4]. The prevalence of lameness in different countries is reported as ranging from 5.1% in Sweden to 54.8% in the North-East of the United States [5–10].

Because of the high prevalence of lameness, its devastating impact on animal welfare and economics, and due to the poor recognition of lame cows by farmers [11–13], various studies focused on automatic lameness detection. The accuracy of weighing platforms [14,15], accelerometers [16–18], combinations of weighing platforms and accelerometers [19–21] or automated video analysis [22,23] were investigated in these studies. However, all the systems presented in these studies are either only applicable on specific farms with corresponding equipment, need additional external data input (e.g. daily milk yield, concentrate intake), are very labor intensive, or lack accuracy [24].

The behavior of lame cows or cows with foot pathologies as compared to healthy, non-lame cows was characterized by longer lying bouts [25], more time spent lying down [25,26] shorter strides [27,28], slower walking speed [19,29,30], lower bite rate while grazing [26], lower feeding time or faster eating (kg/min) [31,32].

Rutten et al. [24] reported that until now, automated lameness detection systems were only able to detect severe lameness, which farmers can easily detect by direct observation. A remarkable amount of economic loss (32%) is caused by foot disorders not associated with any lameness [4], and the prognosis of foot lesions was found to be negatively correlated with the duration of the disease process [13,33]. Therefore, Rutten et al. [24] suggested that automated lameness detection systems would ideally be designed to detect the disease early, allowing disorders of the locomotor system to be treated sooner.

Automated measurement systems have to be valid, reliable and specific on an extended set of behavioral variables as a prerequisite for high accuracy of lameness detection [34,35]. RumiWatch noseband sensors [36,37] and the RumiWatch three-dimensional (**3D**)-accelerometers [35] with the novel algorithm fulfill these criteria. The accelerometers allow for a differentiation of walking versus standing behavior with a high accuracy and further provide an accurate measurement of stride variables (i.e. stride distance and duration) [35]. These are unique features that–to the best of our knowledge–are not offered by any other accelerometer currently available on the market. Therefore, the goal of this study was to evaluate the suitability of the combination of the 3D-accelerometer and the noseband sensor (RumiWatch, ITIN + HOCH GmbH, Fütterungstechnik, Liestal, Switzerland, http://www.rumiwatch.ch/) as an automated lameness monitoring system for dairy cows kept in a cubicle barn. It was hypothesized that the high accuracy of the newly developed RumiWatch algorithms in detecting behavioral variables can be used to develop a lameness detection model with high sensitivity and specificity.

## Material and Methods

### Ethical Standard

The study was approved by the responsible Thuringian (Germany) State Office of Consumer Protection under the registry number 15-105/14 (Thüringer Landesamt für Verbraucherschutz, Bad Langensalza, Germany).

### Cows and Housing

The study was performed between November 2014 and September 2015 on a dairy farm with 1,003 lactating German Holstein cows in Thuringia, Germany (50°45'24.2"N 11°57'52.9"E). Cows were kept in a cubicle barn. The feeding and walking alleys were covered with plain asphalt and the cubicles (130 cm x 250 cm) with chalk dusted hard rubber mats (2 cm thick). Cows were fed a total mixed ratio ad libitum twice daily (containing grass- and corn-silage, hay, beet pulp, concentrate and minerals), and water was freely available in self-filling troughs. Cows were milked 3 times a day, in a 60-place rotary milking parlor. The mean milk yield per cow (305-days) was 10,492 kg ± 3,602 kg (mean ± SD; fat: 4.35% ± 0.76%; protein: 3.54% ± 0.39%). The cows’ claws were routinely trimmed approximately 3 times a year by a professional claw-trimmer.

### Experimental Procedure

**Fig 1:**
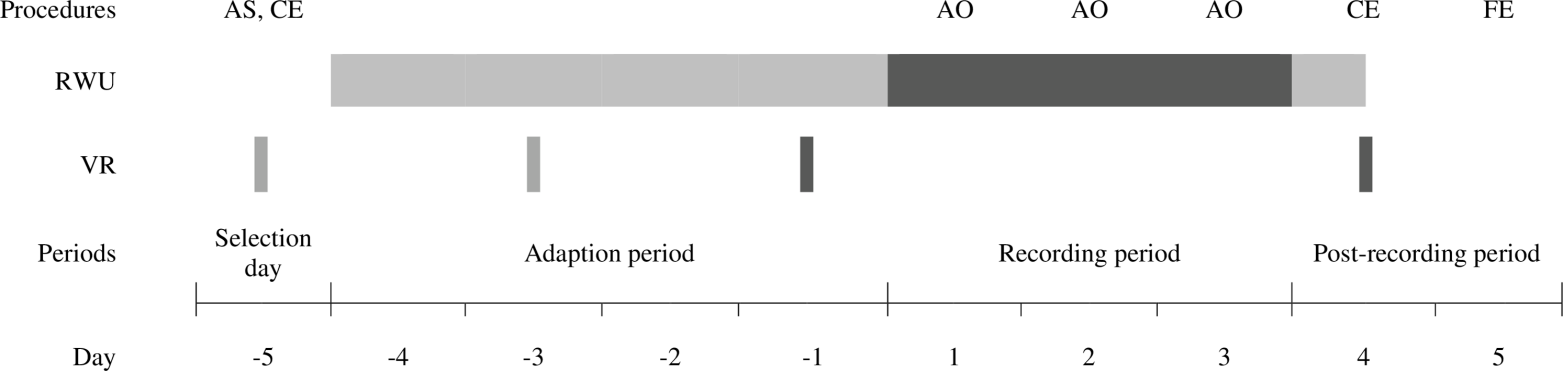
Schematic representation of the experimental procedure per study group. Accelerometers and noseband sensors (RumiWatch-units = RWU) are either attached (light grey) or attached and data recorded (dark grey). Video recording (VR) procedures used for habituation of study cows to the procedure are marked in light grey, VR used for lameness assessment are marked in dark grey. AS = Animal Selection, CE = clinical examination, AO = animal observation (heat, illness, gait scoring), FE = foot examination in the trimming chute.

Experimental procedures were conducted similarly for all cows as described in Fig 1. The cows always entered the study in groups of 4 cows. These groups are herein after referred to as “study groups”. Fifteen study groups successively entered the study over a time period of 11 months.

#### Selection day

At selection day (day -5), study animals were purposely selected by the first author from one of two high-yielding groups according to following selection criteria: At the time of selection, 1 cow per study group was not lame (numerical rating system (**NRS**) according to Flower and Weary [38] ≤ 2) and 3 were lame (NRS ≥ 2.5), whereas only cows that showed lameness located in one or both hind feet, were included in the study. The selection criteria for study animals were: daily milk yield > 25kg in the previous week, not pregnant for longer than 6 months and parity 2 to 5. Cows were not included if suspected of any systemic disease during clinical examination, if any anti-inflammatory drugs had been administered within 28 days prior to selection, or if they were within the withdrawal period for any antibiotics. Corresponding data were retrieved from the herd management program (HERDE 5.8, dsp-Argrosoft GmbH, Ketzin/Havel, Germany, http://www.dsp-agrosoft.de/). Each cow’s gait was video recorded with a digital camera (Nikon Coolpix L830, Nikon Corporation, Tokyo, Japan, http://www.nikon.com/), and a clinical examination, including estimation of body condition score (**BCS**), body weight (**BW**) and measurement of withers height (**WH**), was performed in the catching feeding fence.

#### Adaptation period

At day -4, each cow included in the study was equipped with 2 3D-accelerometers to each hind limb, attached proximal to the fetlock joint and one halter including a noseband sensor (accelerometer and halter = RumiWatch units = **RWU**) as described by Alsaaod et al. [35] and Ruuska et al. [37], respectively. Thus, cows were familiarized with the RWU for at least 3 days. At day -3 and day -1, video recordings of each cow’s gait were made.

#### Recording period

The recording period lasted for 3 days from day 1 to day 3. RWU were checked daily for proper function and were replaced, if necessary. Each replacement of RWU took place in the milking parlor in order not to move the animals additionally. The farm staff was instructed not to unnecessarily manipulate study cows (e.g. claw trimming during recording period, barn group changing). In order to detect heat, the paper based heat table used by the farm staff was checked. In addition, the first author at least once a day directly observed whether cows showed any signs of heat or systemic disease. Once daily, cows were gait scored to detect any extraordinary variation of lameness during the recording period.

#### Post-recording period

At day 4, video recordings of the cow’s gait were performed, as well as a second clinical examination, including BCS, BW and WH. RWU were detached and data were saved. The following day (day 5), a thorough examination of the feet was performed in the claw trimming chute.

### Data Collection

#### Clinical examination

All organ systems were clinically examined according to Dirksen [39] at day -5 and day 4. The cows were not considered healthy in presence of: urine ketone ≥ ++ Ketostix, (Bayer AG, Leverkusen, Germany), rectal temperature (**RT**) > 39.5°C, lesions proximal to the feet causing lameness, lameness located in the front limbs, swollen and painful udder, purulent vaginal discharge, gastrointestinal disorders, cardiac murmurs, severe infection of respiratory tract or nervous disorders. If any cow was considered not to be healthy during any of the research periods, it was excluded from the experiment.

BW was estimated using a measuring tape according to Yan et al. [40]. To estimate body condition, Edmondson’s BCS [41] was determined. WH was measured at the level of the forelimb, using a meter ruler with a horizontal rod (including a spirit level). For BCS, BW and WH, the means of the 2 measurements were taken for further analysis. As BCS is recorded on a quarter point scale, the mean value was rounded up to the next quarter.

#### Lameness scoring

Cows were videotaped walking up and down an asphalt covered passageway (2 m x 30 m) in front of the camera. A handler walked immediately behind the cows, encouraging them to walk, if necessary. All video recordings were made 2 to 4 hours after milking at day -5, day -3, day -1 and day 4. The NRS of each cow was determined using video recordings of day -1 and day 4. Each cow’s gait was independently rated by 3 experienced (at least one year of experience) observers (GB; MA; AdS), resulting in 6 lameness scores per cow. Video recordings were rated in random order, in order to blind observers. Recordings with scores deviating for more than 1 point among the 3 observers were independently rated once again. The mean of the 6 scores was calculated, rounded to the nearest 0.5 point and used for further analysis for the particular animal. Cows with an NRS ≤ 2 were classified as non-lame (group C) and cows with an NRS ≥ 2.5 as lame (group L). Group L was subdivided into three subgroups, which were defined as follows: mildly lame (group LI; NRS = 2.5–3), moderately lame (group LII; NRS = 3.5) and severely lame (group LIII; NRS ≥ 4).

#### Foot examination

Foot examination was performed at day 5 in a claw trimming chute (Klauenpflegestand Defi HY 300, Defi Woldegk GmbH, Woldegk, Germany). Each limb was lifted, cleaned and photographed. Pathologies were documented by using dedicated software [42] (Klauenmanager 1.5, S.E.G. Informationstechnik GmbH, Bad Ischl, Austria, http://www.klauenmanager.eu/). Functional claw trimming and, if necessary, treatment of foot disorders were undertaken by a professional claw trimmer. Foot pathologies were classified according to the ICAR Claw Health Atlas (http://www.icar.org/Documents/ICAR_Claw_Health_Atlas.pdf), cases of digital dermatitis were sub-classified as acute lesions (i.e. M2 according to Berry et al. [43]) or other stages of digital dermatitis (i.e. M1, M3, M4 and M4.1 according to Berry et al. [42]).

#### Feeding and locomotion behavior

After the recording period was completed (day 4), raw data were transferred via USB cable from RWU to a personal computer using a specialized software (RumiWatch Manager 2, Version 2.1.0.0, ITIN + HOCH GmbH, Liestal, Switzerland, http://www.rumiwatch.ch/). Raw data were then converted into 1-hour-summaries (S1 Table) using the novel converter developed by Alsaaod et al. [35] for 3D-accelerometers and Zehner et al. [36] for noseband sensors, respectively, and then converted into 24-hour-summaries (S2 Table) using R-script (R-script “Summary Zeitperioden Zusammenfassung”, Innoclever, Liestal, Switzerland, http://www.innoclever.ch/). R-script calculated the sum of all variables within one day, except for the variables, “chews per minute”, “chews per bolus”, “stride duration” and “stride distance”, the means of which were weighted by “ruminating time”, “bolus” and “strides”, respectively. Weighted means were calculated using following formula:

$$\frac{\sum_{n=1}^{24}\left(a_{n}\cdot x_{n}\right)}{\sum_{n=1}^{24}\left(x_{n}\right)}$$

whereas *n* = recorded day hour, *a* = variable of which the mean is to be weighted (e.g. stride distance) and *x* = variable which *a* is weighted by (“ruminating time”, “bolus” or “strides”).

Days during which cows showed symptoms of heat, were inseminated or obviously ill were discarded. If more than one day had to be discarded, we excluded the animal from the study. For statistical analysis, 24-hour-summaries of 2 days (if data of only 2 days was available) or 2 randomly selected days (if data of 3 days was available) and one randomly selected accelerometer, were merged in an Excel spreadsheet. Means and weighted means, respectively, of the two days were taken, resulting in averaged 24-hour-summaries (S3 Table). The variable “calculated walking speed” (walking speed_calc_; m/s) was calculated by dividing “stride distance” (cm) by “stride duration” (ms) and multiplying by 10. The variable “lying bout duration” (min) was calculated by dividing the variable “lying time” (min) by the variable “lying bouts”. Table 1 lists all RWU variables and the definitions used in this study; a “standing bout” is defined as a period, a cow is in upright position but not walking.

**Table 1:** Variables of RumiWatch noseband sensors and 3D-accelerometers (RumiWatch, ITIN+HOCH GmbH, Fütterungstechnik, Liestal, Switzerland).

Variable	Definition
Halter	
Eating time^a^	Eating time per day in min
Ruminating time^a^	Ruminating time per day in min
Eating chews^a^	Number of eating chews per day
Ruminating chews^a^	Number of ruminating chews per day
Bolus^a^	Number of rumination boluses per day
Chews per minute^a^	Number of rumination chews per rumination minute
Chews per bolus^a^	Number of rumination chews per rumination bolus
Accelerometer	
Lying time^b^	Lying time per day in min
Standing time^b^	Standing time per day in min
Walking time^b^	Walking time per day in min
Lying bouts^b^	Number of lying periods > 50 s per day
Standing bouts^b^	Number of periods in a not walking upright position per day. If changing position from lying to standing the period must last > 50 s. If changing from walking to a standing upright position the period must last > 4 s
Walking bouts^b^	Number walking periods per day with at least 3 consecutive strides. Time between 2 strides must not exceed 4 s
Lying bout duration^c^	Mean daily lying bout duration in minutes, “lying time” / “lying bouts”
Strides^b^	Number of strides within walking bouts per day
Stride duration^b^	Mean daily stride duration in ms
Stride distance^b^	Mean daily stride distance in cm
Walking speed_calc_^c^	Mean daily walking speed in m/s, 10 • “stride distance” / “stride duration”

^a^variable validated by Zehner et al. [36].

^b^variable validated by Alsaaod et al. [35].

^c^proportion of validated variables.

### Analysis and Statistics

All statistics were performed by analyzing the averaged 24-hour-summaries of the two randomly selected days using NCSS8 (NCSS, LLC, Kaysville, Utah, USA, http://www.ncss.com/). Initially, a total of 15 study groups containing 15 healthy and 45 lame German Holstein cows entered the study. However, 7 cows had to be excluded, due to heat (n = 1), lameness treatment (n = 1), obvious illness (mastitis (n = 1), hock infection causing a not feet dependent lameness (n = 1)) and loss of RWU-data (n = 3). Therefore, averaged 24-hour-summaries of 12 healthy and 41 lame cows were included in the statistical analysis.

For comparison between group C and group L, the equal-variance T-test and the Aspin-Welch unequal-variance test for normally distributed variables with equal and unequal variance, respectively, were used. For not normally distributed variables the Wilcoxon rank sum test was used. To compare between different locomotion scores (group C, LI, LII, and LIII), the ANOVA and the Kruskal-Wallis test for normally and not normally distributed data, respectively, were used. ANOVA *P*-values were corrected for multiple testing using Bonferroni correction. The Kruskal-Wallis multiple-comparison Z-value test (Dunn's test) was used to determine statistically significant differences between groups, where the Z-value after Bonferroni correction was used. For all tests a *P*-value < 0.05 was considered as statistically significant.

All variables that were significantly different in the T-test, Aspin-Welch-test and Wilcoxon test, respectively, between lame and non-lame cows were analyzed for their ability to predict lameness using univariable logistic regression models. To determine sensitivity and specificity of the model prediction at a given cutoff, a receiver operating characteristic (**ROC**)-analysis was performed. Statistically significant variables were then included in a multivariable logistic regression model. Only variables not correlated with each other (Spearman correlation coefficient > -0.5 and < 0.5) were combined in the same model. Variables were eliminated from the model by stepwise backward selection. In addition, the ROC analyses before and after removing a variable were compared to determine how much the variable added to the sensitivity and specificity.

## Results

### Cows

Cows from groups L and C did not differ (*P* > 0.05) in parity, BCS and BW. However, group L cows were older (*P* < 0.05), had more days in milk (**DIM**; *P* < 0.05), a lower daily milk yield (**DMY**; *P* < 0.01), a higher WH (*P* < 0.01) and a lower RT (*P* < 0.05) than cows from group C. Group L (n = 41) had a mean NRS of 3.35 ± 0.95 (mean ± SD), ranging from 2.5 to 4.5, while Group C (n = 12) had a NRS of 1.75 ± 0.34 (mean ± SD), ranging from 1 to 2. In group L, 19 cows were assigned to group LI (NRS = 2.5–3), 11 cows to group LII (NRS = 3.5) and 11 cows to group LIII (NRS ≥ 4). Detailed information is provided in Table 2.

**Table 2:** Clinical variables of non-lame (group C) and lame (group L) cows.

	Group C (n = 12)^a^				Group L (n = 41)^b^				
Variable^c^	Mean	SD	Median	IQR^d^	Mean	SD	Median	IQR^d^	*P*-Value
Parity	2.58	0.67	2.50	1.00	2.88	0.95	3.00	1.50	0.4303
Age	4.07	0.61	4.04	0.86	4.72	1.23	4.64	1.90	0.0172
DIM	98.21	42.57	86.75	84.38	153.13	87.90	146.50	107.75	0.0310
DMY	47.10	8.01	51.45	12.76	39.75	7.56	39.80	9.40	0.0051
BCS	3.13	0.27	3.25	0.44	2.87	0.42	2.75	0.63	0.0509
WH	147.92	2.83	148.00	4.63	151.52	3.88	151.00	5.50	0.0043
BW	650.05	29.97	655.27	52.02	655.59	48.61	656.23	77.67	0.7109
RT	38.71	0.18	38.7	0.35	38.54	0.25	38.6	0.3	0.0290
NRS	1.75	0.34	2.00	0.50	3.35	0.59	3.50	1.00	<0.0001

^a^Group C: Non-lame = Numerical rating system (NRS) according to Flower and Weary [38] ≤ 2.

^b^Group L: lame = NRS ≥ 2.5.

^c^Variable: Age in years; DIM = Days in milk at recording period; DMY = Daily milk yield at recording period; BCS = Body condition score according to Edmondson et al. [41]; WH = withers height; BW = estimated body weight according to Yan et al.[40]; RT = rectal temperature in degrees Celsius at selection day; NRS = Numerical rating system according to Flower and Weary [38].

^d^Interquartile range.

### Foot Pathologies

We found one or more foot pathologies in 52 of 53 study animals. Cows from group C (n = 12) showed one or more of the following foot pathologies on at least one claw or foot: interdigital dermatitis (67%; n = 8), moderate or severe heel horn erosions (50%; n = 6), circumscribed sole hemorrhages (33%; n = 4), acute digital dermatitis lesion (i.e. M2 according to Berry et al. [43]; 8%; n = 1), double sole (8%; n = 1), thin sole (8%; n = 1), and white line abscess (8%; n = 1).

Cows from group L (n = 41) showed one or more of the following foot pathologies on at least one claw or foot: interdigital dermatitis (61%; n = 25), sole ulcers (44%; n = 18), moderate or severe heel horn erosions (44%; n = 18), circumscribed sole hemorrhages (37%; n = 15), double soles (34%; n = 14), thin soles (29%; n = 12), white line abscesses (29%; n = 12), diffused sole hemorrhages (22%; n = 9), interdigital phlegmons (7%; n = 3), interdigital hyperplasia (7%; n = 3), toe ulcers (5%; n = 2) and acute digital dermatitis lesion (2%; n = 1).

### Feeding Behavior

Data of feeding behavior of both groups are given in Table 3. Cows from group L spent less (*P* < 0.001) time eating and did fewer (*P* < 0.01) eating chews compared to non-lame cows (mean ±SD; 301.93 ± 57.16 min/day vs. 378.61 ± 71.40 and 22,320 ± 4,780 vs. 29,270 ± 7,210, respectively). Likewise, ruminating time (537.37 ± 59.63 min/day vs. 583.23 ± 56.28; *P* < 0.05), ruminating chews (36,900 ± 5,780 vs. 40,760 ± 4,190; *P* < 0.05) and ruminating boluses were fewer (533.96 ± 120.76 vs. 614.50 ± 60.74; *P* < 0.05) in group L cows as compared to group C cows. Chews per minute and chews per bolus did not differ between the two groups (*P* > 0.1).

**Table 3:** Variables of RumiWatch noseband sensors and accelerometers (RumiWatch, ITIN+HOCH GmbH, Fütterungstechnik, Liestal, Switzerland) of non-lame (group C) and lame cows (group L).

	Group C^a^				Group L^b^				
Variable	Mean	SD	Median	IQR^c^	Mean	SD	Median	IQR^c^	*P*-Value
Halter									
Eating time, min/d	378.61	71.40	364.10	128.01	301.93	57.16	297.38	80.53	0.0003
Ruminating time, min/d	583.23	56.28	582.77	81.56	537.37	59.63	552.09	71.30	0.0327
Eating chews, 1000/d	29.27	7.21	28.24	13.29	22.32	4.78	22.05	6.94	0.0074
Ruminating chews, 1000/d	40.76	4.19	41.46	7.45	36.90	5.78	37.80	7.10	0.0363
Bolus, 1/d	614.50	60.74	615.25	109.25	533.96	120.76	550	100.25	0.0173
Chews per minute, 1/min	75.74	4.70	74.63	7.08	73.97	5.65	75.13	5.29	0.7580
Chews per bolus, 1/bolus	65.87	3.80	66.88	5.38	69.85	13.91	67.57	6.02	0.4765
Accelerometer									
Lying time, min/d	679.65	74.13	692.92	45.60	784.38	130.56	803.01	187.25	0.0059
Standing time, min/d	718.97	73.64	707.88	46.00	618.13	128.68	607.92	179.83	0.0087
Walking time, min/d	41.74	5.99	40.38	9.10	37.85	7.06	37.25	11.62	0.0892
Lying bouts, 1/d	9.79	1.60	9.75	2.00	9.54	3.35	9.00	3.75	0.2675
Standing bouts, 1/d	119.63	17.17	118.25	24.88	97.91	17.73	96.50	25.25	0.0004
Walking bouts, 1/d	111.17	16.28	111.50	22.38	89.79	16.97	86.50	24.00	0.0003
Lying bout duration, min/bout	71.72	18.30	70.53	17.96	91.06	31.40	88.62	43.24	0.0363
Strides, 1/d	1075.17	151.51	1023.00	235.00	950.82	176.15	939.00	236.50	0.0200
Stride duration, s/stride	1.83	0.84	1.80	0.14	1.97	0.15	1.98	0.24	0.0002
Stride distance, m/stride	1.31	0.14	1.31	0.17	1.06	0.16	1.09	0.23	<0.0001
Walking speed_calc_, m/s	0.72	0.09	0.74	0.12	0.54	0.10	0.55	0.12	<0.0001

^a^Group C: Non-lame = Numerical rating system (NRS) according to Flower and Weary [38] ≤ 2.

^b^Group L: lame = NRS ≥ 2.5.

^c^Interquartile range.

### Locomotion Behavior

Data of locomotion behavior of both groups are given in Table 3. Cows from group L spent more time lying down (784.38 ± 130.56 min/day vs. 679.65 ± 74.13 min/day; *P* < 0.01) and less time standing (618.13 ± 128.68 min/day vs. 718.97± 73.64 min/day; *P* < 0.01), than group C cows. Lying bout duration was longer in group L than in group C (90.06 ± 31.40 min/bout vs. 71.72 ± 18.30 min/bout; *P* < 0.05). Walking time was not significantly different, although group L cows tended to spend less time walking, than group C cows (37.85 ± 7.06 min vs. 41.74 ± 5.99 min; *P* < 0.1). We observed no difference in the number of lying bouts between group L and group C. However, the number of walking bouts (89.79 ± 16.97 vs. 111.17 ± 16.28 bouts per day; *P* < 0.001) and consequently the number of standing bouts (97.91 ± 17.73 vs. 119.63 ± 17.17 bouts per day; *P* < 0.001) was lower in group L than in group C. Likewise, the number of strides was lower in group L than group C (950.82 ± 176.15 vs. 1,075.17 ± 151.51; *P* < 0.05).

Cows from group L had longer lasting (*P <* 0.001) and shorter (*P <* 0.0001) strides than group C cows. Mean stride duration and stride distance was 1.97 ± 0.15 s and 1.06 ± 0.16 m, respectively, for group L cows, compared to 1.83 ± 0.84 s and 1.31 ± 0.14 m for group C cows, respectively. Consequently, the walking speed_calc_ was lower in group L than in group C (0.54 ± 0.1 m/s vs. 0.72 ± 0.09 m/s; *P <* 0.0001).

**Fig 2:**
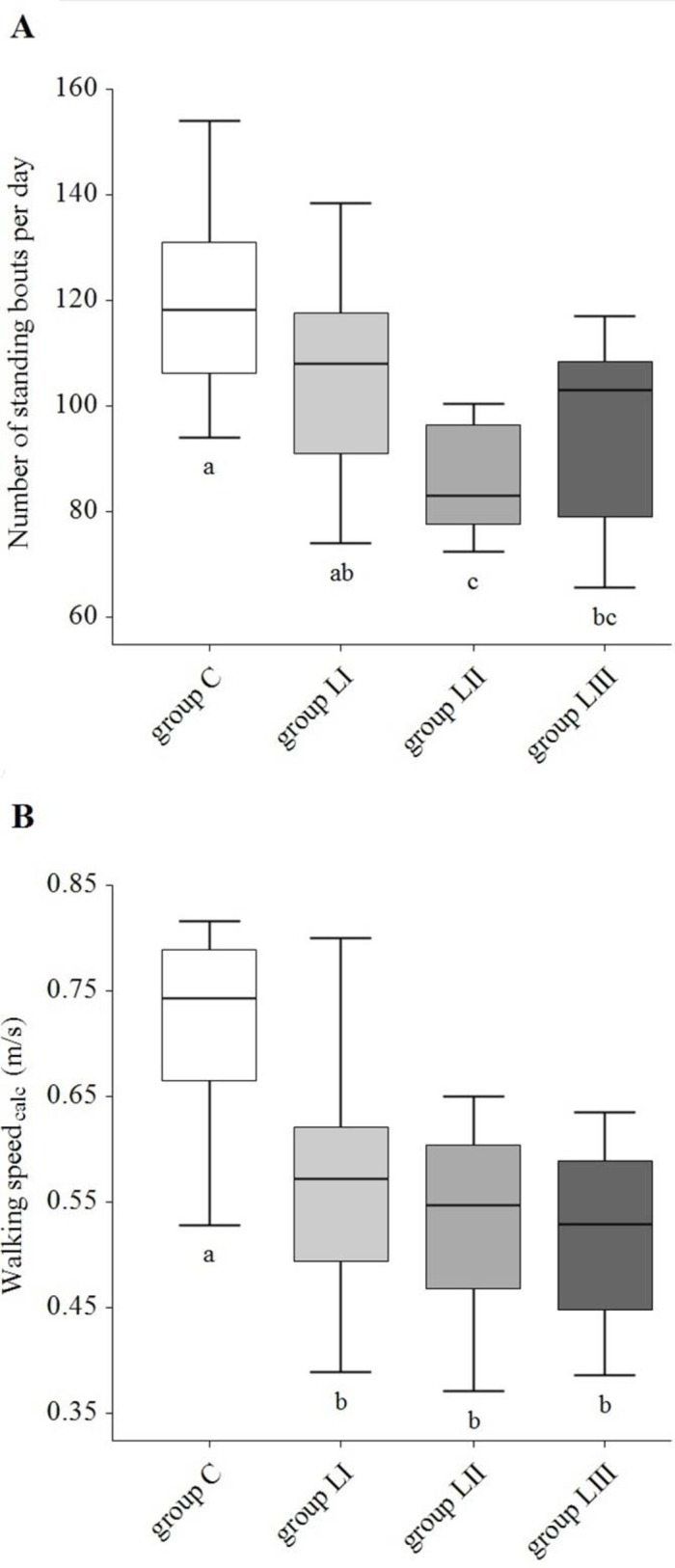
**Box Plot representations of number of standing bouts (A) and walking speed_calc_ (B) of group C (NRS ≤ 2), group LI (NRS = 2.5–3), group LII (NRS = 3.5) and group LIII (NRS ≥ 4).** Different characters (a, b, c) indicate a significant difference (*P* < 0.05). Numerical rating system (NRS) is according to Flower and Weary [38].

Comparison of group C cows with groups LI–LIII, revealed differences for “stride distance” (*P <* 0.001) and “walking speed_calc_” (*P <* 0.0001), whereas the three lameness groups LI–LIII did not differ among each other (Fig 2A). Standing and walking bouts were different (P < 0.0001), although not between group C and group LI, but between group C and groups LII and LIII (Fig 2B).

### Logistic Regression

**Fig 3:**
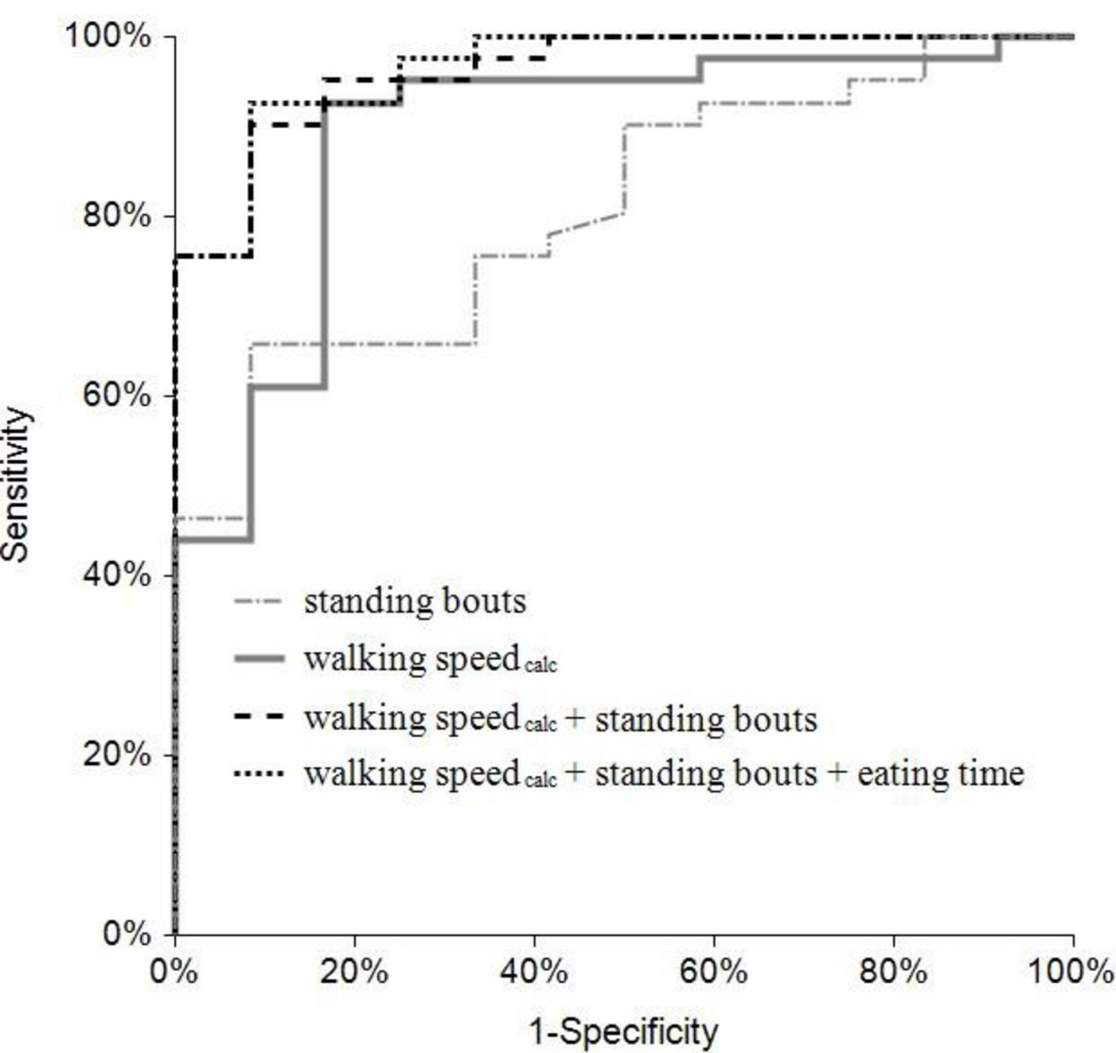
Receiver operating characteristics (ROC) curves of different logistic regression models to discriminate between lame (NRS ≥ 2.5) and non-lame cows (NRS ≤ 2). First model (-∙-) includes standing bouts (AUC = 0.81), second model (─) includes walking speed_calc_ (AUC = 0.88); third model (- -) includes walking speed_calc_ and the number of standing bouts (AUC = 0.96); fourth model (∙∙∙) includes walking speed_calc_, the number of standing bouts and the eating time (AUC = 0.96). NRS = numerical rating system according to Flower and Weary [38]. AUC = area under the receiver operating characteristics curve.

Results of univariable logistic regression models are shown in Table 4. The multivariable logistic regression models with the best model fit are shown in Table 5. ROC-curves of the univariable models of “walking speed_calc_” and “standing bouts”, the multivariable model of “walking speed_calc_” and “standing bouts” and the multivariable model of “walking speed_calc_”, “standing bouts” and “eating time” are depicted in Fig 3.

**Table 4:** Results of univariable logistic regression and receiver operating characteristics analysis of a cow being lame (numerical rating system according to Flower and Weary [38], NRS ≥ 2.5) using different RumiWatch noseband sensor and accelerometer (RumiWatch, ITIN+HOCH GmbH, Fütterungstechnik, Liestal, Switzerland) variables as predictors on the cutoff value with highest sensitivity + specificity.

Variable	OR^a^	95%CI	*P*_Wald_	AUC^b^	R-squared	Cutoff	Sensitivity,%	Specificity, %	Proportion Correct, %
Halter									
Eating time^c^	0.55	0.37–0.82	0.0032	0.79	0.22	297.4	51.2	100.0	62.3
Ruminating time^c^	0.61	0.39–0.95	0.0290	0.72	0.11	577.1	73.2	66.7	71.7
Eating chews^d^	0.81	0.71–0.93	0.0033	0.78	0.21	29,849.0	97.6	50.0	86.8
Ruminating chews^d^	0.84	0.71–1.00	0.0445	0.70	0.09	42,888.5	92.7	41.7	81.1
Bolus^e^	0.91	0.83–0.99	0.0355	0.73	0.11	600.5	80.5	66.7	77.4
Accelerometer									
Lying time^c^	1.27	1.04–1.54	0.0184	0.77	0.12	754.6	63.4	100.0	71.7
Standing time^c^	0.79	0.65–0.96	0.0205	0.76	0.12	661.7	68.3	91.7	73.6
Standing bouts^e^	0.50	0.32–0.80	0.0033	0.81	0.22	104.0	65.9	91.7	71.7
Walking bouts^e^	0.50	0.32–0.78	0.0025	0.82	0.22	97.5	73.2	91.7	77.4
Lying bout duration^c^	2.38	0.98–5.82	0.0567	0.70	0.08	77.0	63.4	91.7	69.8
Strides^f^	0.67	0.46–0.99	0.0423	0.72	0.08	958.0	58.5	83.3	64.2
Stride duration^g^	2.42	1.26–4.65	0.0083	0.78	0.17	1,857.6	75.6	75.0	75.5
Stride distance^h^	0.35	0.19–0.64	0.0008	0.88	0.35	122.6	90.2	83.3	88.7
Walking speed_calc_^i^	0.18	0.07–0.45	0.0003	0.88	0.41	0.65	92.7	83.3	90.6

^a^Odds ratio.

^b^Area under the receiver operating characteristics curve.

^c^OR adjusted to an increase of 30-min; Cutoff: time in minutes.

^d^OR adjusted to an increase of 1000 chews; Cutoff: absolute number of chews.

^e^OR adjusted to an increase of 10 boluses/bouts; Cutoff: absolute number of boluses/bouts.

^f^OR adjusted to an increase of 100 strides; Cutoff: absolute number of strides.

^g^OR adjusted to an increase of 0.1-sec; Cutoff: stride duration in ms.

^h^OR adjusted to an increase of 10-cm; Cutoff: stride distance in cm.

^i^OR adjusted to an increase of 0.1 m/s; Cutoff: walking speed_calc_ in m/s.

**Table 5:** Using different RumiWatch noseband sensor and accelerometer (RumiWatch, ITIN+HOCH GmbH, Fütterungstechnik, Liestal, Switzerland) variable combinations as predictors of a cow being lame (numerical rating system according to Flower and Weary[38], NRS ≥ 2.5) in multivariable logistic regression and receiver operating characteristics analysis on different cutoff-values with corresponding sensitivity and specificity.

Model	OR^a^	95% CI^a^	*P*_Wald_^a^	R-squared^b^	AUC^b^	Cutoff^c^	Sensitivity, %^d^	Specificity, %^d^	Correctly classified, %^d^
Walking speed_calc_	0.12	0.03–0.49	0.003						
+ Standing bouts^e^	0.89	0.81–0.98	0.014	0.61	0.96	-0.06	90.2	91.7	90.6
						1.77	75.6	100.0	81.1
						-2.08	100.0	58.3	90.6
Walking speed_calc_	0.15	0.04–0.62	0.008						
+ Standing bouts	0.90	0.82–0.99	0.026						
+ Eating time^f^	0.99	0.97–1.01	0.396	0.62	0.96	-0.49	92.7	91.7	92.5
						2.09	75.6	100.0	81.1
						-2.08	100.0	66.7	92.5

^a^Odds ratio, 95% confidence interval and *P*_Wald_ of the variables within the model.

^b^R-squared and area under the receiver operating characteristics curve of the model.

^c^Cutoff-value after application of model equation

^d^Sensitivity, Specificity and correctly classified study animals of the model on corresponding cutoff-value.

^e^Model equation: 25.6859 - (0.1143 • “standing bouts”)—(20.9763 • “walking speed_calc_”)

^f^Model equation: 26.3199 - (0.0091 • “eating time”)—(0.1043 • “standing bouts”)—(18.8167 • “walking speed_calc_”)

Even though most univariable models show a significant association between the respective RWU-variable and lameness, they have sensitivity or specificity of less than 80%. The univariable models of “stride distance” and “walking speed_calc_”, however did account for 35% and 41% of the variation in the likelihood of a cow being lame (R² = 0.35; R² = 0.41), with an area under the ROC-curve (**AUC**) of 0.88 each, a sensitivity of 90.2% and 92.7%, respectively, and a specificity of 83.3%, each. The univariable models of the variables “standing bouts” and “walking bouts”, had an AUC of 0.81 and 0.82, respectively, and an R-squared of 0.22 each, and high specificity (91.7%) in detecting lame cows, but a rather low sensitivity (65.9% and 73.2%, respectively).

When we combined different variables within one model we were able to achieve an additional increase in prediction quality. The model considering the variables “standing bouts” and “walking speed_calc_” was the best predictor for cows being lame, when accelerometer variables only were used. It explains 61% of the variation in the likelihood of a cow being lame (R² = 0.61), with an AUC of 0.96, a sensitivity of 90.2% and a specificity of 91.7%. Adding additional variables only slightly improved the prediction. The model with highest accuracy in lameness prediction in our study animals is the model considering the data of “walking speed_calc_”, “standing bouts” and “eating time”. It explains 62% of the variation in the likelihood of a cow being lame (R² = 0.62), with an AUC of 0.96, a sensitivity of 92.7% and a specificity of 91.7%.

## Discussion

The results of this study show that lame cows differ in a broad set of behavioral variables from, non-lame cows. Cows from group L were identified with high sensitivity (90.2%) and specificity (91.7%) using data of 3D-accelerometers only. Additional use of the noseband sensor improved the model quality by a 2.5%-increase in sensitivity. The model taking data of the walking speed_calc_, the number of standing bouts and the eating time had the highest sensitivity and specificity.

In order to minimize the seasonal effects (environmental temperature, humidity, light), management (day time of milking, walking distance to milking parlor) and feeding upon the lame and non-lame cows, we selected all cows from one study group out of the same pen group and included 1 non-lame and 3 lame cows in each study group. As group L includes cows with different degrees of lameness, group L cows were expected and proven during this study to have higher variance for most measured variables (Table 3) than group C cows. Therefore, a higher sample size for lame than for non-lame cows was chosen [44].

Data analysis was performed with averaged 24 hour summaries of two days. As our goal was to evaluate the suitability of RWU for early lameness detection, we regarded 48 hours as a short enough time period. The use of longer time periods would delay the lameness detection as a day where a particular cow would have been detected as lame would have had less weight in the mean of multiple days. The use of only 24 hour summaries, on the other hand, would have been more prone to day to day variation and outlier days.

We randomly selected one of the two 3D-accelerometers for data analysis in order to be close to real life practice conditions. The threshold of the NRS for group L was set at 2.5. This is purposely lower than in other studies (NRS ≥ 3 [25,45] or NRS > 3 [15,19]). Cows with impaired locomotion do not always show all traits of a particular locomotion score [46,47]. The score developed by Flower and Weary [38] allows the use of half-integer scores if a cow exceeds the traits of a particular score, but does not meet all of the following score. As our goal was to define a set of variables allowing for the detection of even slight lameness through early detection, we regarded a cow with an NRS of 2.5 as lame, because it met some of the NRS 3 criteria. In order to decrease prevalence of several claw lesions a previous study recommended claw treatment of cows with a locomotion score > 1 [48], using a similar scoring system [49]. We sought for an early detection of lameness, because foot lesions have a better prognosis, the earlier they are treated [13,33].

Comparing group L with group C revealed no differences concerning parity, BCS and BW. Differences between lame and non-lame cows in this study were evident for age, DIM, DMY, WH and RT. Because the main selection criterion was the presence or absence of lameness, these differences can be explained through selection bias for these variables. Differences were expected for DMY [50], are in the physiological range for RT, are small and therefore most likely not biologically relevant for age and WH and were inevitable due to farm management for DIM.

It is widely recognized that some foot pathologies are not associated with increasing locomotion score or lameness [4,5,10,51–53]. Our results of the feet examination support these findings. Group C cows showed various foot disorders, primarily interdigital dermatitis and heel horn erosion which are usually not associated with lameness [4,54]. Therefore, automatic detection of foot disorders using accelerometers might be more difficult than automatic detection of lameness. However, since our goal was to automatically detect lameness (i.e. NRS ≥ 2.5) and not to automatically detect foot disorders, this hypothesis needs further proof in subsequent studies.

Lameness was significantly associated with a wide range of feeding and locomotoion variables. Lame cows spent less time feeding and were also found to eat faster [31,32], thus reducing time standing in order to minimize pain [32]. Our results support this hypothesis, as eating time and number of eating chews, as well as standing time, were significantly lower in lame cows. As a consequence, lame cows spent more time lying down also confirming results of previous studies [25,26,30].

The number of lying bouts did not differ between lame and non-lame cows, a result also found in other studies [21,25]. The fact that lame cows are less willing to rise, seems only to be reflected in lying time and lying bout duration. The number of standing bouts in our study is highly correlated (r = 0.98; *P* < 0.0001) with the number of walking bouts, as a new standing bout is counted, for every non-walking upright position after a walking bout (Table 1). To our knowledge, no other study investigated the effect of lameness on the number of walking bouts. In order to minimize the time in upright position, lame cows mainly have walking bouts with a specific purpose, for example to get to the next feeding place. This explains the difference in the number of walking and standing bouts between group C and group L. Regarding the large differences in the number of standing and walking bouts and the high specificity in the univariable logistic regression models, we conclude that these variables can be useful for automatically detecting lame cows, especially when they are combined with other accelerometer derived variables.

The difference in number of strides was significant, yet very small, not allowing for sufficient discrimination between lame and non-lame cows. Chapinal et al. [20] reported a similar situation regarding the number of steps in one study and no difference in number of steps at all in another study [19]. However, a direct comparison between the variable “strides” in our study and the variable “steps” in their studies is difficult because “stride” is narrowly defined as a forward or backward movement of the limb within a walking phase only [35]. The minor difference in our study might well exist, because every cow has to walk the same distance to the feeding fence and milking parlor (most strides are associated with milking [20]), requiring a minimal number of strides for each cow, regardless of the lameness status. Additionally, lame cows did take shorter strides requiring more strides to travel the same distance, similar to healthy cows on slippery floors [55]. Likewise, the walking time did not differ between lame cows and non-lame cows, because lame cows take longer to walk a given distance (e.g. milking parlor). Stride duration and walking speed_calc_ were lower in group L, supporting this hypothesis. Our results of walking time are in accordance with Hassal et al. [56], who also reported no difference in walking time in cows held on pasture. In other studies, a significant difference in walking time was found between non-lame and lame cows [26,57]. However, one study [26] investigated estrus behavior in lame and healthy cows on pasture and in the other study [57], cows during estrus or diseased cows were not excluded. Therefore, a comparison is difficult.

Similar to our study, earlier research also showed that lame cows and cows with claw pathologies, respectively, had shorter strides [27,28,58], longer lasting strides [27] and a lower walking speed [19,28–30,58]. Interestingly, the calculated walking speed in our study was a better predictor of a cow being lame, accounting for 41% of the variation (R^2^ = 0.41) and an AUC of 0.88, than the walking speed assessed by video recordings in the study of Chapinal et al. [19], accounting for 22% of variation (R^2^ = 0.22) and an AUC of 0.73, despite the less strict lameness definition in their study (NRS > 3 vs. ≥ 2.5). This may be explained by the fact that Chapinal et al. [19] measured the walking speed in an artificial setting (walking down an alley, while encouraged to walk by a person) while in our study, walking speed was calculated from variables that were collected throughout the whole recording period. Assessing mean daily walking speed using accelerometers is a practical and non-invasive method (i.e. no need to walk behind the cow to encourage walking). Our results suggest that walking speed measured by RumiWatch 3D-accelerometers is a promising variable for automated lameness detection.

Logistic regression results indicate that RumiWatch 3D-accelerometers alone provide a sufficient accuracy in predicting lameness in cows. To our knowledge this is the first lameness detection model with a sensitivity and a specificity over 90% using accelerometers only. The very high correlation of accelerometer variables with visual observation [35] did contribute to the high accuracy in lameness detection. In this study, we included cows with different degrees of lameness, and the model also performed well in the detection of cows with mild or moderate lameness. Because the additional use of the noseband sensor is more expensive, the data do not substantially improve the models, and eating time was not significant within the multivariable logistic regression model (Table 5), we do not a priori recommend the use of the predictive model including the variable eating time.

Animal behavior depends on management conditions. Therefore, our threshold values cannot be regarded as global standard values of the respective variables. Stride distance is known to differ on different floor types [28,59]. The number of strides and the lying time [20] and most likely the number of walking and standing bouts, the walking time and lying bout duration, depends on milking frequency, the milking system (i.e. automated milking system vs. milking parlor) and distance to the milking parlor. Moreover, we only included multiparous German Holstein cows in this study. Primiparous cows are more active than multiparous cows [60,61]. Often primiparous cows are not full-grown, possibly affecting stride distance [28,59]. Also the interactions with estrus and disease, presumably affecting sensitivity and specificity, respectively, were not investigated. Furthermore, the study was conducted in a cross-sectional study design, where behavioral parameters of non-lame cows were compared to those of cows with varying degrees of lameness. The results therefore merely capture the ability of the halters and accelerometers to distinguish between non-lame and lame cows at a given time. Due to the study design, one cannot make assumptions on how well the system performs in detecting changes in individual cows during the transition from non-lame to lame. Still, the results of this pilot study are very promising and show the potential of the RumiWatch system for lameness detection, warranting longitudinal studies on this topic, allowing identifying transition from a non-lame to a lame status.

## Conclusions

The results of this study show that the RumiWatch 3D-accelerometers with the novel algorithm for detection of extended locomotion characteristics and noseband sensors are able to detect differences in behavior in lame and non-lame cows. Models accounting for two 3D-accelerometer variables only (walking speed_calc_, standing bouts) automatically identified lame cows (NRS ≥ 2.5) with great accuracy. We, therefore, conclude that the RumiWatch-system may be suitable for lameness detection, even of slight lameness. Management factors influence the behavior of dairy cows. Thus, a multicenter longitudinal study is needed to validate the results of our study in various farms under different management conditions and to capture transitions between different states of locomotion.

## Supporting Information

S1 Table One-hour summaries. Table of one-hour summaries of all animals and days included in the study.
(XLSX)

S2 Table Twenty-four-hour summaries. Table of 24-hour summaries of all animals and days included in the study.
(XLSX)

S3 Table Data used for statistics. (XLSX)
